# Could natural phytochemicals be used to reduce nitrogen excretion and excreta-derived N_2_O emissions from ruminants?

**DOI:** 10.1186/s40104-023-00942-0

**Published:** 2023-11-09

**Authors:** Yuchao Zhao, Ming Liu, Linshu Jiang, Leluo Guan

**Affiliations:** 1 Beijing Key Laboratory of Dairy Cow Nutrition, College of Animal Science and Technology, Beijing University of Agriculture, Beijing, 102206 China; 2 College of Animal Science and Technology, China Agricultural University, Beijing, 100193 China; 3 Department of Agricultural, Food and Nutritional Science, University of Alberta, Edmonton, AB T6G 2R3 Canada

**Keywords:** Nitrogen metabolism, Nitrous oxide, Plant bioactive compounds, Ruminant, Urine patches

## Abstract

Ruminants play a critical role in our food system by converting plant biomass that humans cannot or choose not to consume into edible high-quality food. However, ruminant excreta is a significant source of nitrous oxide (N_2_O), a potent greenhouse gas with a long-term global warming potential 298 times that of carbon dioxide. Natural phytochemicals or forages containing phytochemicals have shown the potential to improve the efficiency of nitrogen (N) utilization and decrease N_2_O emissions from the excreta of ruminants. Dietary inclusion of tannins can shift more of the excreted N to the feces, alter the urinary N composition and consequently reduce N_2_O emissions from excreta. Essential oils or saponins could inhibit rumen ammonia production and decrease urinary N excretion. In grazed pastures, large amounts of glucosinolates or aucubin can be introduced into pasture soils when animals consume plants rich in these compounds and then excrete them or their metabolites in the urine or feces. If inhibitory compounds are excreted in the urine, they would be directly applied to the urine patch to reduce nitrification and subsequent N_2_O emissions. The phytochemicals' role in sustainable ruminant production is undeniable, but much uncertainty remains. Inconsistency, transient effects, and adverse effects limit the effectiveness of these phytochemicals for reducing N losses. In this review, we will identify some current phytochemicals found in feed that have the potential to manipulate ruminant N excretion or mitigate N_2_O production and deliberate the challenges and opportunities associated with using phytochemicals or forages rich in phytochemicals as dietary strategies for reducing N excretion and excreta-derived N_2_O emissions.

## Introduction

Ruminant animals are extremely important not only for producing the highest quantity of milk and meat as essential parts of human diets, but also for their ability to feed on fibrous feeds that cannot be used as human food [1]. However, 75%–90% of consumed nitrogen (N) is excreted as urine and feces [2]. As the excreted N exceeds the plant demand, it can result in considerable N losses via nitrate (NO_3_^−^) leaching, ammonia (NH_3_) volatilization, and nitrous oxide (N_2_O) production [2]. N_2_O, a potent greenhouse gas (GHG), is estimated to be 298 times more powerful than carbon dioxide (CO_2_) in warming power over 20 years [3]. By oxidizing into nitrogen oxides in the stratosphere, N_2_O plays a vital role in depleting stratospheric ozone [4]. Anthropogenic N_2_O emissions (2.7 ± 1.6 GtCO_2_-eq) were 133% higher in 2019 than in 1990 [3]. Approximately 81% of anthropogenic N_2_O emissions are attributed to the agricultural sector [3], and N_2_O emissions from ruminant excreta account for 46% of agricultural N_2_O emissions [5].

Ruminant production is projected to continue growing in the next few decades, driven by fast growth of human population, rising incomes, and dietary preferences towards ‘Western’ diets [6]. Given the growing concerns over the environmental impacts of ruminant farming, there is an impetus to decrease the emissions of ruminant-derived N_2_O substantially. Over the years, several manure N and N_2_O mitigation strategies, including dietary or manure management approaches at the herd level along with more targeted approaches, such as reducing dietary protein intake [7], supplementing sodium chloride [8], changing rumen undegradable protein sources [9], alternative forage [10, 11], and nitrification inhibitors [12, 13] have been widely investigated. Contemporary consumer demands orient towards the use of ‘natural products’ to alter ruminant N metabolism and excreta-derived N_2_O emissions.

On the one hand, regulating the N metabolism of ruminants using nutritional strategies could decrease N excretion and, consequently, reduce N_2_O emissions. Previous studies indicated that the addition of natural phytochemicals (e.g., tannins, saponins, and essential oils) could reduce ruminal NH_3_ concentration and alter excreted N partitioning, which helps to lower urinary N excretion and N_2_O emissions to the atmosphere [14–16]. On the other hand, phytochemicals may lessen N_2_O loss when they are deposited in urine, either as diuretics to lower pasture N loading rates or as natural nitrification inhibitors because of their antibacterial activities [17, 18]. Inhibitory substances found in forage can be applied directly to urine patches after being consumed by ruminant livestock. For example, *Plantago* aucubin and *Brassica* isothiocyanates have been shown to inhibit a crucial step in N_2_O generation from urine patches in ruminants fed these forages [19–21].

However, the role and efficacy of phytochemicals in reducing N_2_O emissions from the excreta of ruminant livestock remain controversial. The reasons for the controversy are multifaceted, mainly including the variable effect of reducing N_2_O emissions, different assessment methods of N_2_O emissions, the source of phytochemicals, the number of phytochemicals ingested, the cost of additional feeding, and possible side effects in terms of performance and health. In this review, the potential, mechanisms, and unsolved problems of reducing N_2_O emissions from ruminant livestock through feeding phytochemicals are also discussed. Our purpose is to provide deeper insights into use of phytochemicals to manipulate N excretion and mitigating N_2_O emission from ruminants.

## N_2_O production from ruminant excreta

**Fig. 1:**
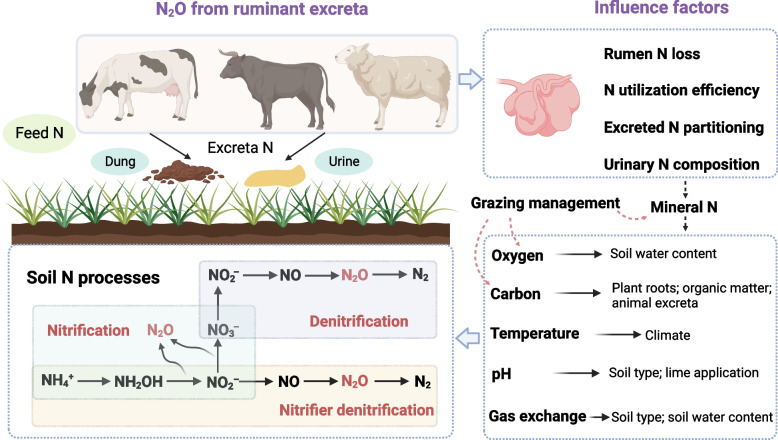
A brief overview of the N_2_O production pathways in ruminant excreta applied to soil and the potential influence factors. *N* Nitrogen

N_2_O is predominantly generated through two major biological pathways, i.e., nitrification and denitrification [22], but may also be produced by other processes such as nitrifier-denitrification or codenitrification [23]. As shown in Fig. 1, following excreta deposition, the major fraction of organic N or urea N in manure is mineralized or hydrolyzed into ammonium (NH_4_^+^) and then converted to NO_3_^−^ via the activity of nitrifiers under partial aeration. The NO_3_^−^ produced can then be transformed into dinitrogen (N_2_) by denitrifying bacteria under anoxic conditions. During nitrification of NH_4_^+^ and denitrification of NO_3_^−^, N_2_O gas may escape into the atmosphere as a by-product. The proportion of N released as N_2_O from ruminant excreta is significantly influenced by feeding regimes, environmental circumstances, farm systems, and manure management practices [24]. We can divide these influencing factors into two facets (i.e., animals and environmental factors) according to the N cycle between ruminants and the environment (Fig. 1).

In the latest review, Mancia et al. [25] discussed the factors of disaggregation of N_2_O emission factors (EF), such as excreta type, animal diet, seasonal variations, and spatial variability. Considering the association between ruminant excreta composition and dietary mitigation strategies, this review focuses on disaggregation by excreta type. In the 2006 Intergovernmental Panel on Climate Change (IPCC) guidelines, the default values of EF were 2% and 1% for cattle and sheep, respectively, with no distinction between urine and dung [26]. The more recent IPCC values of N_2_O EF have been updated, and the urine-based and dung-based N_2_O-EF for cattle was 0.77% and 0.13%, respectively, in a wet climate [27]. Most studies reported that the N_2_O-EF of urine patches were greater than the dung-based N_2_O-EF [28–32], except the results depicted by Wachendorf et al. [33] and Ma et al. [34].

Compared with a longer time for dung N mineralization, urine could rapidly supply available NH_4_^+^ for nitrification and denitrification by hydrolyzing urea, contributing to higher N_2_O losses [35]. Additionally, the high dry matter (DM) content of the dung also reduced the potential for dung N to infiltrate into the soil, restricting interaction with the soil microbial community [36]. The difference implies that the ratio of dung N to urine N excreted can also influence the amounts of N_2_O emissions. The benefit of disaggregating emissions into dung and urine is that the effect of diet manipulation on N partitioning and thus on N_2_O emissions can be accounted for. Therefore, the disaggregation by excreta type offered a better opportunity to regulate N_2_O emissions through dietary phytochemicals.

## Potentials of natural phytochemicals in reducing N excretion and N_2_O emissions

Diet has a profound effect on the chemical composition and partitioning of excreted N, and may therefore indirectly affect N_2_O emission from excreta patches [37]. Several phytochemicals (e.g., tannins, essential oils, saponins, and glucosinolates) present in forages and plant extracts have been identified as possible methane (CH_4_) inhibitors in the rumen [38–41]. Nonetheless, there are numerous opportunities to simultaneously reduce N waste and CH_4_ production in ruminants [15]. In this section, recent advances using dietary phytochemicals as N excretion and N_2_O emission mitigation strategies are described here.

### Tannins

#### Condensed tannins

**Fig. 2:**
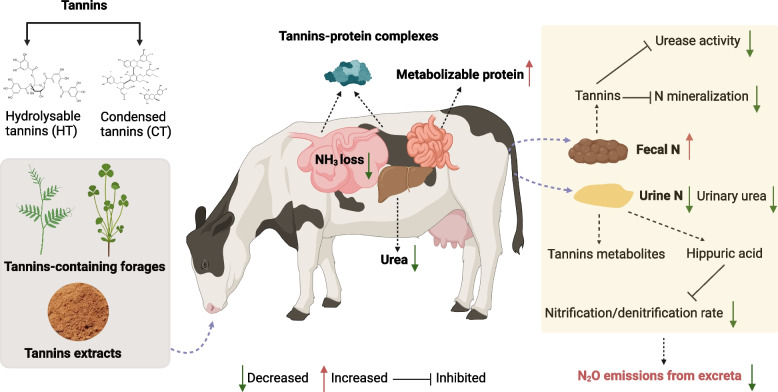
The model of action of dietary tannins in reducing N excretion and N_2_O emissions from ruminat excreta. *N* Nitrogen

Conventionally, tannins are usually classified into two groups: condensed tannins (CT) and hydrolyzable tannins (HT). CT or proanthocyanidins are formed via C4–C8 and C4–C6 interflavonoid connections between chatequins, leucoanthocyanidins, and their derivatives; CT is not rapidly degraded in the gastrointestinal tract [42]. CT can interact with plant proteins via hydrogen bonding in the near neutral pH range to form insoluble tannin-protein complexes, which are subsequently dissociated in the abomasum to release protein. CT may hinder the growth of proteolytic microbes, thereby inhibiting proteolysis [43]. Therefore, the formation of tannin-protein complexes and the suppression of microbial proteolytic activity may reduce rumen degradability and increase the flow of protein into the intestine [44] (Fig. 2).

Numerous studies have reported that dietary supplementation with CT reduced urinary N output and increased fecal N excretion in dairy cows [45], beef cattle [46], sheep [47], lambs [48], and goats [49] (Table 1). The shift in N excretion from urea in the urine to a more stable form of N in feces can increase soil organic N concentration and reduce N_2_O emissions [50]. A field study demonstrated that feeding beef steers with a tannin‑rich legume (sericea lespedeza hay) effectively reduced the emission of N_2_O, CH_4_, and CO_2_ from the soil for 32 d after the application of manure [51]. However, Hao et al. [52] reported that supplementing CT in the diet of beef cattle did not affect composted manure N_2_O emissions. The converse effects were obtained by de Souza et al. [53], who observed that *Acacia mearnsii* tannin extract elevated N_2_O emissions from excreta patches in the field; however, this unexpected result cannot be well explained. These findings suggested that the effects of CT on ruminant N utilization and N_2_O emissions can be highly diverse depending on origin, concentration, molecular structure, and tannin dosage.

**Table 1:** Effects of dietary condensed tannins or forages containing condensed tannins on nitrogen metabolism and N_2_O emissions from excreta in ruminants

Reference	Tannin source (plant/extract)	Animal species	DMI, production performance, or N metabolism related indicators	N excretion or N retention	N_2_O emissions
Grainger et al. [54]	*Acacia mearnsii*	Grazing dairy cows	–DMI; ↓Milk yield	↓UN; ↑FN; –N retention	NR
Deaville et al. [47]	Mimosa tannins	Wether sheep	↓DMI	↓UN; ↑FN; –N retention	NR
Hao et al. [52]	*Acacia mearnsii*	Beef cattle	NR	NR	–Manure N_2_O emissions
Maamouri et al. [55]	*Acacia cyanophylla* foliage	Grazing ewes	↓DMI; ↓Milk yield; ↓MUN	↓UN; –FN; ↑NUE	NR
Kronberg and Liebig [56]	Quebracho	Grazing sheep	–DMI; ↓BUN	↓Urinary urea N	NR
Ahnert et al. [57]	Quebracho	Heifers	–DMI; ↓CP digestibility	↓UN; ↑FN; ↑N retention	NR
Min et al. [58]	*Pinus taeda* L	Meat goats	–DMI; –CP digestibility;	–UN; –FN	NR
Aguerre et al. [59]	Quebracho-chestnut tannin extracts	Dairy cows	↓DMI; –Milk yield; ↑Milk/DMI; ↓CP digestibility; ↓Rumen NH_3_-N; ↓MUN	↓UN; ↑FN; –NUE	NR
Gunun et al. [60]	*Antidesma thwaitesianum* Muell. Arg	Goats	–DMI; –CP digestibility; –Rumen NH_3_-N	↓UN; –FN; ↑N retention; ↑NUE	NR
Pathak et al. [48]	*Ficus infectoria* and *Psidium guajava* leaf meal mixture	Lambs	↑DMI; ↑ADG; ↑FCR; ↑Wool yield; –CP digestibility	↓UN; ↑FN; ↑N retention; ↑NUE	NR
Gerlach et al. [45]	*Acacia mearnsii*	Dairy cows	–DMI; –Milk yield; –Milk composition; ↓MUN	–UN; –FN; ↓NUE	NR
Koenig et al. [46]	*Acacia mearnsii*	Beef cattle	–DMI; –ADG; –FCR; –Carcass traits; ↓BUN	↓Manure NH_3_-N emissions	NR
Koenig and Beauchemin [61]	*Acacia mearnsii*	Beef cattle	–DMI; ↓CP digestibility; ↓BUN	↓Urinary urea; ↓UN; ↑FN	NR
Zhang et al. [62]	Bayberry/*Acacia mangium*	Dairy cows	–DMI; –Milk yield; –Milk composition; ↓MUN; ↓CP digestibility (*Acacia mangium*); ↓BUN (bayberry)	↑FN (*Acacia mangium*); ↓UN (bayberry); ↑N retention (bayberry); ↓N retention (*Acacia mangium*);	NR
Lagrange et al. [63]	Birdsfoot trefoil and sainfoin	Grazing beef cattle	↓BUN	↓Urinary N concentration; ↓Urinary urea-N concentration	NR
de Souza et al. [53]	*Acacia mearnsii*	Sheep	–DMI; ↓CP digestibility	↑FN; – UN; –N retention	↑Urine N_2_O emissions; –Feces N_2_O emissions
Silveira Pimentel et al. [49]	*Acacia mearnsii*	Goat kids	–DMI; ↓FCR; ↓Carcass yield; ↓CP digestibility	↓UN; ↑FN; –N retention	NR
van Cleef et al. [51]	Sericea lespedeza	Beef steers	NR	NR	↓Urine N_2_O emissions; ↓Feces N_2_O emissions; ↓Urine N_2_O-EF; ↓Feces N_2_O-EF;
da Silva Aguiar et al. [64]	*Mimosa tenuiflora* hay	Lambs	↓DMI; ↓CP digestibility	↓UN; ↑FN; –N retention	NR
Uushona et al. [65]	*Acacia mearnsii*	Lambs	↑DMI; ↓CP digestibility; ↑Rumen NH_3_-N	↑FN; –UN; ↑N retention; ↑NUE	NR
Oliveira et al. [66]	*Acacia mearnsii*	Dairy cows	–DMI; –Milk yield; –Milk composition; ↓Milk UFA	–UN; –FN	NR

*DMI* Dry matter intake, *MUN* Milk urea nitrogen, *BUN* Blood urea nitrogen, *CP* Crude protein, *ADG* Average daily gain, *FCR* Feed conversion ratio, *NH*_*3*_*-N* Ammonia nitrogen, *UN* Urinary nitrogen, *FN* Eecal nitrogen, *NUE* Nitrogen utilization efficiency, ↑ = Increase, ↓ = Decrease, – = No statistically significant effect, *NR* Not reported

However, most studies in the past decades investigating the effects of dietary CT on N excretion were conducted with penned ruminant livestock under intensive feeding systems. Only a few articles have been published regarding the effects of fresh forages rich in tannins on excreta-derived N gaseous losses under year-round grazing. *Acacia cyanophylla* foliage containing 3% CT fed to grazing ewes up to 200 g/d reduced urinary N excretion and increased NUE [55]. Lagrange et al. [63] found that a combination of tanniferous legumes (birdsfoot trefoil and sainfoin) led to reductions in urine N and urinary urea-N concentration that were larger than the decrease observed for the single tanniferous species alone. However, it remains controversial whether tannins can maintain their biological activity during the haymaking process [67]. Stewart et al. [15] reported that feeding CT-containing hays [birdsfoot trefoil (0.6% CT) or sainfoin (2.5% CT)] or HT-containing hay [small burnet (4.5% HT)] to Angus heifers or beef cows also reduced urinary urea N excretion and shifted the partitioning of N from urine to feces, compared to feeding traditional legume and grass hays. The results of Stewart et al. [15] suggested that tannins retain their biological capabilities (i.e., influencing N metabolism) regardless the modifications during the haymaking stage. Furthermore, the potential of adding chestnut and mimosa tannin to grass at ensiling to improve N utilization in sheep was also investigated by Deaville et al. [47], who found that compared to the control silage, both tannins decreased urinary N excretion and increased fecal N output.

By mixing CT into the feed of penned ruminant livestock, enough CT intake can be achieved; however, when cattle and sheep are grazing, it can be difficult to achieve an adequate and consistent consumption of a feed supplement containing CT. Kronberg and Liebig [56] showed that supplementing quebracho tannins to the freshwater of grazing sheep lowered urine urea deposition onto grasslands, and evaluating the feasibility of adding CT to drinking water to minimize N_2_O emissions from urine patches in pastures is warranted. Tannins have been studied extensively in reducing N or N_2_O emissions in confined livestock raised in temperate climates. However, the use of tannins can be extended to other production systems by considering their basic biology.

In addition to reducing of urine N_2_O emissions through decreasing urinary urea excretion, the CT presented in feces may provide a feasible strategy to reduce N_2_O emissions by applying manure (a combination of dung and urine) to agricultural soils. Over 50% of CT remains undigested in the ruminant gastrointestinal tract [68, 69]. In this light, it should be no surprise that ruminants fed a diet high in CT will excrete CT-rich feces. Tannins from birdsfoot trefoil were shown to be present and potentially active in the feces of dairy cows, as shown by Misselbrook et al. [70]. When dairy calves were fed dietary CT, NH_3_ emissions from slurries on the barn floor [70] and slurries applied to soil [70, 71] were reduced. Powell et al. [71] observed that urease activity in feces and NH_3_ emissions from manure were both reduced when dairy cattle were fed chestnut tannin extract or simulated barn floors were applied with tannins. Recent studies confirmed that N_2_O emissions were reduced from tannin-enriched manure [72, 73].

Additionally, because mineralization of the complex is inhibited, the tannins-protein complexes in feces are more resistant to breakdown in the soil and decompose more slowly than feces without CT [74, 75]. Fagundes et al. [76] reported that feeding *Acacia mimosa* tannin extract to cattle increased fecal N output, delayed organic matter breakdown, and changed soil microbial dynamics following feces application. However, these researchers did not quantify N_2_O emissions from the feces of ruminant fed-CT. Larger-scale studies are required to determine the effectiveness of dietary tannin extracts in abating N_2_O loss from ruminant barn floors and land-applied excreta.

#### Hydrolyzable tannins

Compared with CT, HT has a weaker affinity for proteins and thus is more easily absorbed by the gastrointestinal tract, increasing potential toxicity to the animal [42, 77]. For this reason, previous studies on the utilization of tannins in ruminant livestock focused on CT instead of HT. Nevertheless, an in vitro investigation revealed no difference between tannin sources in preventing protein degradation [78]. Therefore, HT can also bind to bacteria, modifying their activity, and to proteins, reducing their breakdown in the rumen and consequently altering N output. Supplementing HT extracted from chestnut at 1%–3% DM in sheep [79] or coupled with CT extract (derived from quebracho) at 1.5% DM in steers [80] decreased the ruminal NH_3_ concentration (Table 2). Chestnut tannins, as a representative HT, have been shown the potential to minimize the environmental impact of ruminants via the N shift from urine to feces [47, 81].

**Table 2:** Effects of dietary hydrolyzable tannins or forages containing hydrolyzable tannins on nitrogen metabolism and N_2_O emissions from excreta in ruminants

Reference	Tannin source (plant/extract)	Animal species	DMI, production performance, or N metabolism related indicators	N excretion or N retention	N_2_O emissions
Deaville et al. [47]	Chestnut	Wether sheep	↑DMI	↓UN; ↑FN; –N retention	NR
Wischer et al. [81]	Chestnut or valonea	Sheep	–DMI; ↓CP digestibility	↑FN; ↓UN; –N retention; –NUE	NR
Wei et al. [82]	GA	Beef cattle	NR	↑FN; –UN; ↓Urinary urea; –N retention	NR
Yang et al. [83]	TA	Beef cattle	↓BUN	↑FN; ↓UN; ↓Urinary urea; ↑Urinary hippuric acid	NR
Aboagye et al. [80]	Chestnut	Beef cattle	–DMI; –ADG; –FCR; ↓Ruminal NH_3_-N; –BUN	↓UN	NR
Bao et al. [10]	GA	Beef cattle	NR	–FN; –UN; –N retention	↓Urine N_2_O emissions
Aboagye et al. [84]	GA, TA, or chestnut	Beef heifers	–DMI; ↓CP digestibility (TA and chestnut); ↓Ruminal NH_3_-N (TA); ↓BUN (GA, TA, and chestnut)	↑FN (TA and chestnut); –UN; ↓Urinary urea N/UN; –N retention	NR
Zhang et al. [62]	Valonia	Dairy cows	–DMI; –Milk yield; –Milk composition; ↓MUN; ↓BUN; ↓CP digestibility	↑FN; –UN; ↓N retention; –NUE	NR
Zhou et al. [7]	TA	Beef cattle	NR	↑FN; ↓UN; ↓Urinary urea; ↑Urinary hippuric acid	↓Urine N_2_O emissions
Herremans et al. [85]	Oak	Dairy cows	↑DMI; –Milk yield; –Milk composition; ↓Milk SFA; ↑Milk UFA; –Ruminal NH_3_-N; –MUN; –BUN; ↓CP digestibility	↑FN; ↓UN	NR
Kapp-Bitter et al. [86]	Chestnut	Dairy cows	–DMI; –Milk yield; –Milk composition; –CP digestibility	↑UN; –FN	NR

*GA* Gallic acid, *TA* Tannic acid, *DMI* Dry matter intake, *MUN* Milk urea nitrogen, *BUN* Blood urea nitrogen, *CP* Crude protein, *ADG* Average daily gain, *FCR* Feed conversion ratio, *NH*_*3*_*-N* Ammonia nitrogen, *SFA* Saturated fatty acids, *UFA* Unsaturated fatty acids, *UN* Urinary nitrogen, *FN* Fecal nitrogen, *NUE* Nitrogen utilization efficiency, ↑ = Increase, ↓ = Decrease, – = no statistically significant effect, *NR* Not reported

Tannic acid is another typical HT. Yang et al. [83] reported that the supplementation of tannic acid to the diet of beef cattle reduced the ratio of urine N to fecal N and modified the concentrations of nitrogenous compounds in the urine. Gallic acid derives from the hydrolysis of specific HT [87]. Feeding gallic acid to beef cattle altered the pattern of N excretion by increasing the ratio of fecal N to urinary N and decreasing the ratio of urinary urea N to urinary N [82]. Subsequently, laboratory incubation trials demonstrated that adding gallic acid or tannic acid to the diet of steers reduced N_2_O fluxes after applying urine to the soil [7, 10].

In contrast to CT, it is believed that HT can be degradable in the rumen [88]. Dietary supplementation with gallic acid decreased urine N_2_O emissions from beef cattle, while urinary N excretion or urea excretion was not influenced [10]. Bao et al. [10] attributed it mainly to the excretion of gallic acid metabolites, such as pyrogallol and resorcinol, which might inhibit the processes of N_2_O production. However, no direct evidence for this proposed mechanism is currently available. Additionally, a greater amount of urinary hippuric acid excretion was observed in beef cattle fed tannic acid [7, 83]. Hippuric acid excretion correlates with polyphenols consumption because polyphenols are the precursor components to hippuric acid formed during rumen fermentation [89]. The antibacterial compound hippuric acid has been considered for its ability to reduce N_2_O emissions. Researchers have found that enhancing the percentage of hippuric acid in the urine caused a decrease in N_2_O fluxes from bovine urine of 50%–54% [90, 91], most likely due to the inhibition of denitrification or a reduction in the ratio of N_2_O to N_2_ [90]. Bertram et al. [92] reported that hippuric acid also partially inhibited soil nitrification. These observations suggest that an elevation in urine excretion of hippuric acid in response to the consumption of biodegradable polyphenols (e.g., HT) may represent a possible N_2_O mitigation strategy. However, conflicting findings have been found in the limited field tests done so far, with no inhibitory effects of hippuric acid on urine N_2_O fluxes [93, 94]. Further research is needed to identify whether supplementing HT to increase the formation of HT metabolites in urine will decrease subsequent N_2_O emissions.

In a word, although tannins have shown good potential in reducing urinary N and N_2_O from ruminant excreta, it does not mean that they can be promoted in livestock production. The nutritional and environmentally sustainable potential of tannins will only be realized when the composition, structure, and biological function of tannins in plant extracts or forages are better defined. Chemical assays should be complemented by measurements of binding capacity to plant proteins and effects on enzyme activity or in vitro digestion, as the relationship between chemical structure and stringency is not well defined. Analytical and experimental efforts will reveal the most effective tannins for expressing temperate legumes by genetic engineering or conventional selection as part of plant breeding projects or for mitigating N_2_O from ruminant excreta utilizing existing forages.

### Glycosides

#### Glucosinolates

**Fig. 3:**
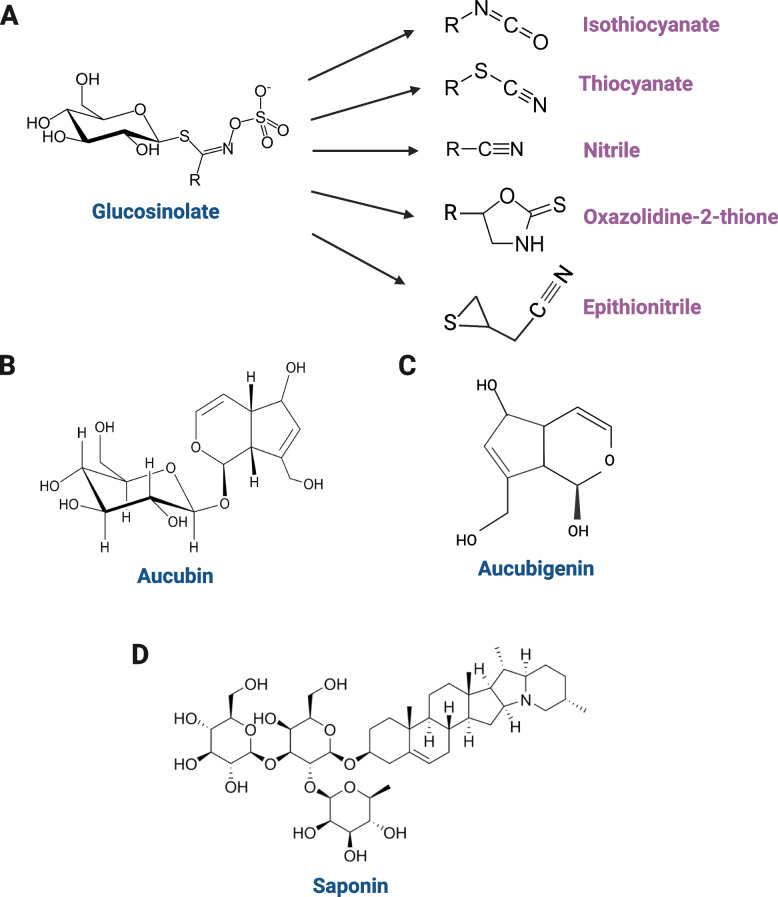
The chemical structure of glucosinolate and its metaboites (**A**), aucubin (**B**), aucubigenin (**C**), and saponin (**D**)

Glucosinolates (GLS) are a large group of plant secondary metabolites with nutritional effects and biologically active compounds. The GLS molecule comprises a β-thioglucose unit, a sulfonated oxime unit, and a side chain derived from an amino acid that varies (Fig. 3A) [95]. There are at least 120 distinct structures of GLS, which are found in 16 families of dicotyledonous plants [95]. Brassica plants are annuals traditionally used to fill feed deficits in temperate ruminant grazing systems [96]. There are five primary degradation products of GLS, with isothiocyanate being the most important, followed by thiocyanate, nitrile, epithionitrile, and oxazolidine-2-thione [97] (Fig. 3A).

The potential of GLS hydrolysis products to act as biological nitrification inhibitors has been investigated. Studies reported that the application of Brassicaceae tissues and subsequent the generation of various GLS hydrolysis products (isothiocyanate, thiocyanate, and nitriles) could weaken the activity of nitrifying bacteria [98] and inhibit nitrification [99]. The GLS hydrolysis products have been shown to inhibit the nitrification processes in soil incubated with brassica seed meals containing GLS [100].

**Fig. 4:**
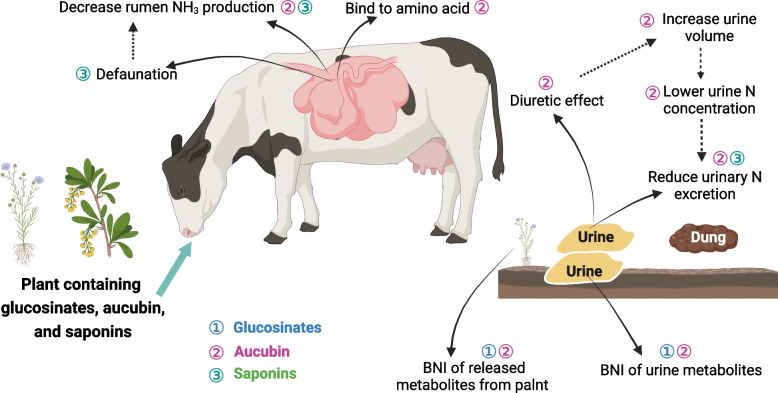
The possible mechanisms for glucosinolates, aucubin, and saponin as ruminant manure N and N_2_O mitigation strategies. *N* Nitrogen, *BNI* Biological nitrification inhibition

GLS and their decomposition metabolites have been detected in the urine of animals fed brassicas [101]. Urine from sheep-fed forage rape (*Brassica napus* L.) had a lower N_2_O-EF than urine from sheep-fed perennial ryegrass (*Lolium perenne* L.) when applied to a freely draining pasture soil in early spring (0.11% vs. 0.27%, respectively) [19] (Table 3). According to Hoogendoorn et al. [102], the urine from sheep-fed ryegrass (*Lolium perenne* L./*Trifolium repens*) had a higher N_2_O EF than sheep-fed fodder rape (*Brassica napus* L.). This decline may be explained by the fact that the brassica-derived chemicals GLS hydrolysis products were transferred from urine to the soil [103, 104], inhibiting nitrification in the urine patch [105]. The promising findings of these experiments indicate that isothiocyanate and other urinary secondary metabolites may function as nitrification inhibitors. In a laboratory study, Balvert et al. [105] demonstrated that several GLS hydrolysis products (isothiocyanate and nitrile) inhibited the nitrification process and lowered N_2_O fluxes from urinary urea applied to soils (Fig. 4). In a field experiment, however, the application of GLS hydrolysis products to artificial urine patches did not result in any significant differences in N_2_O emissions [105].

**Table 3:** Effects of dietary glycosides or forages containing glycosides on nitrogen metabolism and N_2_O emissions from excreta in ruminants

Reference	Glycoside source (plant/extract)	Animal species	DMI, production performance, or N metabolism related indicators	N excretion or N retention Angus heifers	N_2_O emissions
Glucosinolates					
Luo et al. [19]	*Brassica napus* L.	Grazing sheep	–DMI	↑N retention; –UN/N intake; ↑FN/N intake	↓Urine N_2_O emissions
Gao et al. [106]	Rapeseed cake	Beef cattle	–ADG	↑FN; –UN; –N retention; –NUE –N retention	↑Urine N_2_O emissions
Aucubin					
Box et al. [107]	Plantain	Grazing dairy cows	↑Milk yield; ↑Milk lactose percentage; ↓MUN	↓UN; –FN	NR
Cheng et al. [108]	Plantain	Grazing dairy heifers	–DMI; ↓BUN;	↓UN	NR
Minnéeet al. [109]	Plantain	Grazing dairy cows	↑DMI; ↑Milk yield; ↑Milk lactose percentage; ↓MUN	↑FN; ↓UN; ↑Milk N	NR
Marshall et al. [110]	Plantain	Grazing dairy cows	–DMI; –Milk yield; –Milk composition; ↓MUN; –Ruminal NH_3_-N	↑FN; ↓UN; ↑Milk N	NR
Ineichen et al. [111]	Plantain	Dairy cows	↑DMI; ↑Milk yield; –Milk composition; ↑MUN; –FCR	↑FN/N intake; –UN/N intake; ↓NUE	NR
Nkomboni et al. [112]	Plantain	Grazing dairy cows	–DMI; ↑Milk protein percentage; ↓MUN; ↓BUN	–Milk N; –NUE	NR
Al-Marashdeh et al. [113]	Plantain	Grazing dairy cows	–DMI; ↑Milk yield	NR	↓N_2_O emissions
Saponins					
Santoso et al. [14]	*Biophytum petersianum*	Goats	↓CP digestibility; ↓Ruminal NH_3_-N; ↑Microbial N supply	–FN; ↓UN; –N retention	NR
McMurphy et al. [114]	*Yucca schidigera*	Steers	–DMI; –CP digestibility; ↑Microbial N supply; –BUN	–FN; –UN; –N retention; –NUE	NR
Guyader et al. [115]	Tea saponin	Nonlactating cows	↓DMI; –Ruminal NH_3_-N	↓N intake; –FN; –UN; –N retention	NR
Guyader et al. [116]	Tea saponin	Dairy cows	↓DMI; ↓Milk yield; –Milk composition; ↓Milk/DMI; –CP digestibility; –Ruminal NH_3_-N	–FN; –UN; ↓milk N; –N balance	NR
Liu et al. [117]	Tea saponin	Dorper crossbred ewe	–DMI; ↑CP digestibility; ↓Ruminal NH_3_-N	↓FN; ↓UN; ↑N retention; ↑NUE	NR

*DMI* Dry matter intake, *MUN* Milk urea nitrogen, *BUN* Blood urea nitrogen, *CP* Crude protein, *ADG* Average daily gain, *FCR* Feed conversion ratio, *NH*_*3*_*-N* Ammonia nitrogen, *UN* Urinary nitrogen, *FN* Fecal nitrogen, *NUE* Nitrogen utilization efficiency, ↑ = Increase, ↓ = Decrease, – = no statistically significant effect, *NR* Not reported

Furthermore, several experiments have also examined the response of N_2_O production to the metabolite thiocyanate. Snyder et al. [118] observed that 2-propenyl isothiocyanate and thiocyanate released from *Brassica juncea* and *Sinapis alba* seed meals hindered bacterial metabolism and that thiocyanate release was the cause of nitrification inhibition in the *Sinapis alba* treatment. Thiocyanate was also detected in the rumen fluid and urine of cattle fed rapeseed meals [106, 119, 120], whereas isothiocyanate and oxazolidine-2-thione remained undetected in the rumen fluids. Thus, thiocyanate may be the primary product of GLS hydrolysis in the rumen, and that the ruminal GLS metabolic route could be distinct from that driven by myrosinase [119]. Contrary to expectations, in a laboratory study, Gao et al. [106] found that the urine N_2_O emissions and N_2_O-EF were increased in steers received the diet containing high GLS-rapeseed cake. A significant correlation was found between urinary thiocyanate content and urine N_2_O fluxes [106]. Additional laboratory incubation confirmed that thiocyanate enhanced the denitrification of soil nitrifiers, which may have led to an increase in N_2_O emissions [121]. These controversial results suggest that GLS hydrolysis products differ in their effects on N transformations. Therefore, more GLS hydrolysis products excreted in ruminant urine should be assessed for their individual or combination effect on N_2_O production from urine.

Unfortunately, except for the publication by Gao et al. [106], no study has investigated the potential for the feeds containing GLS and the subsequent effect of the voided urine to reduce soil nitrification and N_2_O emissions. Before the intake of forage rich in GLS or their hydrolysis compounds can be perceived as a strategy for decreasing N_2_O emissions, the composition and concentration of GLS hydrolysis products in urine must be determined. More research into the N_2_O production mechanisms in soils treated with urine from ruminants-fed brassica plants under various environmental situations is required. It should be noted that the action of GLS depends on their activity in fertilizing the soil with excreta (mainly urine). However, its activity is often affected by the environment and is full of uncertainties. At the same time, GLS is one of the common antinutritional factors in ruminant feed. Therefore, targeting GLS as an inhibitor to reduce N_2_O emissions may be difficult.

#### Aucubin

Aucubin, an iridoid glycoside found in plantain (*Plantago*), displays similar inhibitory properties as GLS and their metabolites (Fig. 3B). Aucubin is known to degrade completely into its instability aglycone, aucubigenin (Fig. 3C), within 4 h in the presence of β-glucosidase [122], and β-glucosidase is known to be present in soils [123]. Aucubigenin can be converted into an unsaturated aldehyde that binds permanently to the nucleophilic side chains of nucleic acids and proteins, due to its potent alkylating characteristics [124]. Numerous experiments showed that dairy cows grazing plantain (*Plantago lanceolata* L.) or mixed pastures containing plantain had a lower N concentration in spot-sampled urine [18, 107–109, 125] (Table 3), and these researchers hypothesized that the overall output of urine N might also be lowered. This can offer an opportunity to utilize plantain to minimize N losses in grazing ruminants. The increased fraction of undegradable N is one of the potential causes of the lower urine N content in cows received plantains. The greater undegraded N content allows more N to pass through the rumen to be digested in the small intestine, where more N is partitioned to milk and feces, and less N is excreted into urine. Minnée et al. [109] showed that feeding dairy cows with a diet containing 45% DM of plantain reduced the amount of urinary N while increasing the amount of N partitioned to milk and feces by 14%.

The second possible mechanism for decreasing urinary N concentration and increasing urine volume is aucubin's diuretic action. O'Connell et al. [126] confirmed this effect by observing that penned sheep fed plantain emitted more urine than those fed ryegrass. Additionally, Navarrete et al. [127] found that aucubin reduced NH_3_ production in vitro and was likely degraded to its active aglycone aucubigenin in the rumen. Therefore, the third possible mechanism for influence of grazing plantain on urinary output could be partly due to aucubin lowering ruminal NH_3_ losses and reducing urea production in the liver [128]. Aucubin, for instance, is antibacterial due to the aucubin aglycone (aucubigenin), which binds to free amino acids, making them unavailable [122]. The escape from the rumen of these aucubigenin-amino acids complexes could also shift the N partitioning from urine to feces by transferring the digestible N fractions to the intestine. Therefore, aucubin and/or aucubigenin in plantain could potentially change the N partitioning in ruminants (Fig. 4).

Furthermore, soils under plantain showed significantly lower NO_3_^−^ concentrations [129], mineralization and nitrification rates [129], lower ammonia oxidizer bacteria abundance [130], and lower N_2_O fluxes [131] than under other plant species. Likely, plantain root-released chemicals (e.g., aucubin) with biological nitrification inhibitory capacity contributed partially to the reduced N_2_O flux observed in the presence of plantain. Luo et al. [20] observed that comparison of N_2_O emissions from cattle urine applied to plantain and to perennial ryegrass, plantain had lower emissions in winter but not in other seasons. This result may be due to differences in concentrations of aucubin or other secondary metabolites as they could have been affected by temperature or other environmental variables [132]. In addition, broad-spectrum antibacterial action has been observed with aucubin, and aucubigenin has been shown to inhibit cytochrome P-450, which may be related to its capacity to limit ammonia oxidation by inhibiting the activity of the soil enzyme ammonia monooxygenase [133, 134]. Thus, aucubin and its derivative aucubigenin are potential biological nitrification inhibitors (Fig. 4).

It is uncertain what form or concentration of aucubin is excreated in ruminant urine because Navarrete et al. [127] did not quantify the residence time of aucubin and/or aucubigenin during ruminal fermentation or identify its fate following ruminal metabolism. Additionally, plantain was the only plant species tested (550 total) whose antibacterial activities were detectable in rabbit urine 8–16 h after feeding [135]. Even though a rabbit is not a ruminant, similar results can be obtained with ruminant urine. Judson et al. [136] found higher soil NH_4_^+^ contents following the application of urine from dairy cows received 100% plantain compared to the urine of cows fed with ryegrass-white clover. However, the two urine sources had comparable total N contents, suggesting that urine from grazing cows fed plantain can inhibit nitrification. Similarly, Simon et al. [21] observed that the increasing consumption of plantain for grazing cows decreased urinary N loading rates and urine N_2_O emissions. Thus, another potential route for the aucubin in plantain to enter the soil would be through the urine of ruminants grazing plantain-based pastures.

For a short period of time following soil application, aucubin may act as a nitrification inhibitor; however, its inhibitory actions seem insufficient to produce substantial reductions in total urine patch N_2_O fluxes [137]. A latest trial revealed that grazing dairy cows on plantain pastures did not lower urine N_2_O fluxes compared to ryegrass-white clover urine when treated at the same N urine rate [138]. It is hypothesized that aucubin degrades swiftly in soils and that the suppressive action of its decomposition metabolites, notably aucubigenin, persist in soils for no more than a few days [137]. Further studies should determine the metabolic pathways of aucubin in soil, quantify aucubin urination ratios, and investigate the impact of aucubin excretion ratios on the inorganic-N dynamics and N_2_O emissions of urine patches.

#### Saponins

Saponins are a large family of amphiphilic glycosides of steroids and triterpenes (Fig. 3D). Saponins is well known for their potential of decreasing rumen CH_4_ production by decreasing both the number and activity of methanogenic microorganisms [139]. Another important effect of saponins in the rumen appears to be to inhibit the protozoa (defaunation) by affecting cell membrane integrity [140]. Ruminal NH_3_ concentrations are reduced when protozoal growth is inhibited, presumably due to depressed rumen degradation of feed protein or turnover of bacterial protein [14]. NH_3_ concentration also will be altered by binding of NH_3_ to compounds like saponin, as noted by Cheeke [141]. Jouany [142] also assumed that urinary N always decreases with defaunation, due to both the decreased NH_3_ concentration in the ruminal fluid and the increased capture of urea N for microbial protein synthesis. Hu et al. [143] showed that the addition of 40 g/kg of tea saponin led to the lowest concentration of rumen NH_3_-N and the maximum microbial protein content in vitro. Overall, it appears that plants or their extracts with high concentrations of saponins may operate as natural rumen manipulators, which can increase the efficiency of microbial protein synthesis and enhance protein flux to the intestine by decreasing microbial protein turnover.

In a review by Wina et al. [144], 14 out of 51 publications indicated that saponins did not affect rumen NH_3_-N content, whereas 17 indicated an inhibitory effect. Supplementation of *Biophytum* aqueous extract, up to 26 mg/kg BW of saponin, decreased rumen NH_3_-N and urinary N output, thereby increasing microbial N supply and retained N as a proportion of N digested in goats [14]. Ramírez-Restrepo et al. [145] reported that adding tea seed saponin reduced blood urea concentration in tropical Brahman cattle. Liu et al. [117] observed that dietary addition with tea saponin decreased rumen NH_3_-N, fecal N, and urinary N excretion, leading to a significant increase in N retention and NUE in Dorper crossbred ewe. These results indicate that saponins may contribute to mitigating N excretion and N_2_O emissions from ruminants. However, it was shown that tea saponin did not modify the N balance or N excretion of lactating cows [116] or nonlactating cows [115]. These discrepancies may be due to variations in the experimental diets and saponin dosages.

To test the effect of saponin extracts or saponin-rich forages on N_2_O emissions from excreta under grazing circumstances, additional animal studies and field experiments are still needed. To achieve sustained beneficial effects of saponins in diets, it is necessary to conduct extensive research on the interactions between saponin chemical structures, dietary nutrition components, and their influence on the rumen microbial ecology. It is essential to identify the most biologically active saponins that inhibit the activity and abundance of protozoa while possibly stimulating beneficial bacteria and fungi. Certain classes of saponins may have toxic effects on the body and must be examined in vivo in long-term studies. If more active saponins can be isolated and identified from plants, or if plant biotechnology techniques can be used to produce target saponin components, the beneficial effects of saponins could be widely exploited in various feeding systems, assuming it can be demonstrated in the future that saponins in ruminants are effective at reducing animal N excretion and excreta-derived N_2_O emissions.

### Essential oils

Aromatic plants can produce essential oils, complex combinations of volatile organic substances. Essential oil can contain up to 60 chemical substances, such as alcohols, aldehydes, hydrocarbons, ketones, esters, and ethers [145]. Essential oils have been demonstrated to possess the ability to affect ruminal protein degradation and amino acid absorption in the small intestines of ruminant livestock. Numerous studies reported that addition with essential oil decreased rumen NH_3_-N concentration in vitro (e.g., Golbotteh et al. [146]; Patra and Yu [147]; Pawar et al. [148]) or in vivo (e.g., Lin et al. [149]; Toseti et al. [150]; Wu et al. [16]). Carrazco et al. [151] also found that feeding essential oils reduced enteric emissions of N_2_O and NH_3_ in mid-lactation dairy cattle. Essential oil can reduce ammonia levels, likely due to direct inhibition of proteolytic and ammonia-producing rumen bacteria [147]. Their antibacterial characteristics are explained by various mechanisms, including chemical structures and physical properties [152]. Essential oils are hydrophobic, partitioning through lipid cell membranes, disrupting their integrity and stability, and resulting in leakage of cell contents [153]. The hydroxyl group and their relative position in the phenolic structures (in the case of thymol and eugenol) were believed to be important attributes that influence the antibacterial properties of essential oil [152].

Reducing ruminal NH_3_ loss and moving more microbial protein to the small intestine can increase tissue N retention, reducing the urinary N excretion and the potential of N_2_O emission from manure application. Wanapat et al. [154] observed that feeding garlic powder containing essential oil at 80 g/d with urea-treated rice straw decreased urinary N excretion and improved N retention of steers (Table 4). Ribeiro et al. [155] showed that supplementing thyme essential oil enhanced N retention and reduced urinary N excretion compared to monensin. Specifically, the latest meta-analysis of the effectiveness of essential oils revealed that N retention was greater in beef cattle that received essential oil [156]. However, these results should be interpreted with caution because of the low number of studies that reported these response variables [156].

**Table 4:** Effects of dietary essential oils or forages containing essential oils on nitrogen metabolism and N_2_O emissions from excreta in ruminants

Reference	Essential oil source (plant/extract)	Animal species	DMI, production performance, or N metabolism related indicators	N excretion or N retention	N_2_O emissions
Wanapat et al. [154]	Garlic powder	Steers	–DMI; –CP digestibility; ↓Ruminal NH_3_-N; ↓BUN	↓UN; –FN; ↑N retention	NR
Wanapat et al. [157]	Peppermint powder/garlic powder	Beef cattle	–DMI; –Ruminal NH_3_-N; –BUN; ↓CP digestibility (peppermint powder)	–UN; –FN; ↑N retention (garlic powder); ↓N retention (peppermint powder)	NR
Tekippe et al. [158]	Cinnamaldehyde and eugenol	Dairy cows	–DMI; –Milk yield; –Milk composition; ↑Feed efficiency; ↑MUN; –CP digestibility	↑UN; –FN; –Milk N; –NUE	NR
Oh et al. [159]	Carvacrol, eugenol and thymol	Dairy cows	–DMI; –Milk yield; –Milk composition; –CP digestibility; –MUN ↑Milk lactose percentage; ↓MUN	–UN; –FN; –Milk N; –NUE	NR
Ribeiro et al. [155]	Thyme (Thymus vulgaris)	Sheep	–DMI; –ADG; –CP digestibility; –Ruminal NH_3_-N	↓UN; –FN; –N retention	NR
Benchaar [160]	Thymol	Dairy cows	–DMI; –CP digestibility; –Ruminal NH_3_-N; –Milk yield; –Milk composition	–UN; –FN; –Milk N; –NUE	NR
Muñoz-Cuautle et al. [161]	Oregano (*Lippia graveolens*)	Lamb	–DMI; –ADG; –FCR; –Meat quality; –Ruminal NH_3_-N	–UN; –FN; –N retention; –NUE	NR

*DMI* Dry matter intake, *MUN* Milk urea nitrogen, *BUN* Blood urea nitrogen, *CP* Crude protein, *ADG* Average daily gain, *FCR* Feed conversion ratio, *NH*_*3*_*-N* Ammonia nitrogen, *UN* Urinary nitrogen, *FN* Fecal nitrogen, *NUE* Nitrogen utilization efficiency, ↑ = Increase, ↓ = Decrease, – = no statistically significant effect, *NR* Not reported

Other trials using lactating dairy cows have also shown that single or combinations of essential oils containing thymol, eugenol, and/or carvacrol have no effect on N utilization [145, 158, 160]. Muñoz-Cuautle et al. [161] found that including oregano essential oil in the diet did not alter urinary N, fecal N, or N retention in meat lambs. Conversely, Tekippe et al. [158] reported that supplementing 525 mg/d essential oils products containing eugenol and cinnamaldehyde enhanced urinary N excretion in dairy cows. Several parameters, such as trial duration, essential oil chemical composition, and dosages, may account for differences in results among in vivo trials. The contradictory results may also be attributable to variations in the kinds and amounts of dietary protein consumed. Some findings indicate that essential oil can inhibit the colonization and/or subsequent degradation of readily degradable substrates, such as starch and protein, thus impacting the metabolism of amylolytic and proteolytic bacteria [162]. All fermentation processes associated with dietary protein degradation and ruminal NH_3_ production require further investigation. Animal production indices should be quantitatively and qualitatively correlated with the effects of increasing dietary protein escape from the rumen. Overall, the most promising essential oils and their effective concentrations and combinations can be evaluated further in vivo experiments to determine the essential oils (dose and combination) that can be applied on farms. Before their use on farms, the positive effects of commercial essential oils on animal performance and the environment must be established due to their high cost.

## Challenges of phytochemicals as manure N and N_2_O mitigation strategies

### The consistency and comparability of study results

Phytochemicals are highly variable depending on many factors, including plant species, growth environment of the plants (e.g., soil composition, temperature, and moisture stress), stage of plant growth, parts of the plants utilized to extract phytochemicals, and phytochemicals extraction or analysis method [163]. However, currently, there is no standard product composition, structure and purity of any commercial plant extracts for use in livestock production. As a result, there are often considerable variations between research results, making it difficult to ascertain the necessary types and dosages. For example, Cobellis et al. [163] summarized the effects of various single essential oils and essential oil blends on ruminal N metabolism in vivo and in vitro experiments and discovered that in vitro and in vivo results are always inconsistent. As shown in Tables 1 and 2, numerous studies showed that feeding tannins increased fecal N output, which is a result of tannins limiting the absorption of N, and suggests that part of the protein-tannins complexes failing to dissolve in the abomasum, resulting in a loss of dietary protein [164]. However, some studies observed that tannins altered only urinary N. Depending on the chemical structures of tannins and proteins, tannin-protein interactions do not always function optimally and account for these variations among studies.

Numerous in vitro studies have documented the possible ruminal NH_3_ mitigation effects of phytochemicals in vitro. However, it is generally accepted that in vitro systems, while excellent for screening for bulk inhibitors, are not very representative of responses in N excretion and excreta-derived N_2_O for live animals. Therefore, it is strongly suggested that in vitro results be confirmed in in vivo experiments. Moreover, future studies should detail the source of phytochemicals, extraction method, chemical composition, purity, and dosage. In vitro experiments and studies of the minimal inhibitory concentration of rumen or soil microbes using pure active compounds can shed light on their action method, reveal their major active components, and assist in establishing an appropriate dosage. A better understanding of structure–activity relationships would be needed to acquire consistent results from phytochemicals on mitigating ruminant manure N and N_2_O emissions.

### The balance between its efficacy and side effects

Despite extensive research conducted in recent years, using phytochemicals in ruminant livestock remains challenging and very limited for side effects. Tannins in the diet can bring significant benefits to ruminant livestock; nevertheless, high dietary contents or CT with the ‘wrong’ compositional features would reduce digestion and utilization of dietary protein and absorption of crucial amino acids by the ruminant [88]. For example, Ahnert et al. [57] found that the ruminal infusion of a moderate level of quebracho tannin extract may significantly shift N excretion from urine towards feces, while high quebracho tannin extract dosages has detrimental effects on crude protein and fiber digestibility. The binding capabilities of tannins may potentially reduce fiber digestibility by inhibiting cellulolytic enzyme or binding to dietary carbohydrates, reducing ruminal turnover rate, and consequently minimize feed intake and animal production performance [59, 165]. Guyader et al. [116] also reported that milk production, DM intake, and feed efficiency of dairy cows were reduced with tea saponin (0.52% DM). Major deleterious effects of GLS ingestion in animals are reduced palatability, decreased growth and production [166]. In addition, nitriles are known to influence the activities of the liver and kidneys. Thiocyanates inhibit the availability of iodine, whereas oxazolidine-2-thione can induce the morphological and physiological alterations in the thyroid [166].

Overall, the challenge is to determine which phytochemical features can reduce N_2_O production from excreta by improving dietary N utilization and/or exerting the biological nitrification inhibitory activities in the urine, without harming animal health, performance, or farmers' profitability. Throughout the production cycle and across several production cycles (for example, dairy cows), the impact of manure N and N_2_O mitigation strategies on animal health, welfare, and reproduction must be examined through long-term research. Long-term experiments are also needed to study the mechanism of adaptation of gastrointestinal microbes and animals to phytochemical. A better understanding of how phytochemical mitigation strategies impact ruminant product composition, shelf life, sensory traits, and consumer perceptions of livestock products is also essential.

### Systematic research methodology

Although saponins and essential oil have the potential to manipulate ruminant N metabolism, N_2_O fluxes from ruminant excreta using field plots with the static chamber method have not been investigated previously. In grazing systems, biologically inhibition of nitrification through dietary manipulation with GLS or accubin has not been extensively investigated. The following step is to determine the effect of dietary phytochemicals more closely on N_2_O emissions and soil N cycles in larger-scale, longer-term experiments that simulate more closely manure management of commercial ruminant production systems.

Accurately estimating emissions from farmers using manure N and N_2_O mitigation options requires an integrated systems approach. Life cycle assessments (LCA) should evaluate the upstream and downstream impacts of mitigation strategies. Meta-analyses are critical to determine the effectiveness of phytochemicals as mitigation protocol. Additionally, phytochemicals have the potential to decrease rumen CH_4_ emissions. However, few studies have examined phytochemicals' use to reduce main GHG (i.e., CO_2_, CH_4_, and N_2_O) on either the herd level or in individual animals. The goal of reducing GHG is undermined if a strategy reduces N_2_O but increases another GHG. If LCA is performed, only then will this be captured. Additionally, dietary manipulations with phytochemicals targeting excreted N or N_2_O reduction are mostly studied in isolation.

## Conclusions and perspectives

Ruminant production systems are significant contributors to global N loss and N_2_O emissions. As demand for high-quality meat and milk products rises, N_2_O emissions and global temperature will continue to increase. Phytochemicals, because of their anti-microbial activity and easy availability, may be promising agents to enhance NUE and reduce the environmental impact of ruminant N_2_O emissions. The proposed mechanisms of N_2_O reduction using natural phytochemicals include inhibiting rumen NH_3_ production, increasing N partitioning into feces relative to urine, the diuretic effect phytochemical of leading to more frequent urination, and biologically nitrification inhibitor function of plant secondary metabolites from root exudation and/or animal’s urine. Present results indicate that the dietary inclusion of tannins could considerably reduce N excretion and excreta-derived N_2_O emissions from cattle excreta, whereas the possible negative effects of tannins on ruminant feed intake and nutrient digestibility are of concern. Compared with tannins, none of the existing studies has provided conclusive evidence of the effectiveness and mechanisms of plant glycosides or essential oils in reducing N excretion and N_2_O emissions, and these compounds should also be evaluated in long-term in vivo trials for their effect on N metabolism and N_2_O production. Thus, further studies are required to determine their bioactive compositions, effective doses, mode of action, effect on animal performance and health, and cost–benefit ratio before phytochemicals can be applied as additives on farms to minimize N excretion and N_2_O emissions from ruminant excreta.

Another major issue is affordability; ruminant farmers need greater information on the cost of natural phytochemicals or forages rich in phytochemicals and their impacts on animal productivity, particularly for concentrated animal feeding operations. Regulatory approval requirements for some promising feed ingredients may slow their adoption, and a lack of consumer acceptance of some of them may preclude their use for N_2_O mitigation. Incentives and low-cost approaches may be needed to encourage adoption because, in most cases, decreased N excretion and N_2_O production have not increased ruminant performance.

Additionally, it can be challenging to assess the system-wide effects of N_2_O reduction practices, even though they may be beneficial at specific stages of the production cycle. Therefore, it is critical to analyze mitigation strategies and procedures based on natural phytochemicals or forages rich in phytochemicals in the context of the whole system and LCA to ensure efficiency gains across all levels. In a word, phytochemicals may have a place in sustainable ruminant production scenarios only if more convincing results of their efficacy and effectiveness in mitigating N excretion and GHG emissions are dependably identified. The old saying “do not put all your eggs in one basket” still applies to phytochemical research.

## Data Availability

Not applicable.

## Abbreviations

CH_4_: Methane
CT: Condensed tannins
DM: Dry matter
GHG: Greenhouse gas
GLS: Glucosinolates
HT: Hydrolyzable tannins
IPCC: Intergovernmental Panel on Climate Change
LCA: Life cycle assessments
N: Nitrogen
N_2_O: Nitrous oxide
NH_3_: Ammonia
NH_4_^+^: Ammonium
NO_3_^−^: Nitrate
NUE: Nitrogen utilization efficiency
